# Supplementary material for: Infection control, occupational and public health measures including mRNA-based vaccination against SARS-CoV-2 infections to protect healthcare workers from variants of concern: A 14-month observational study using surveillance data
Source: PLoS One. 2021 Jul 16;16(7):e0254920. doi: 10.1371/journal.pone.0254920 (PMC8284646; doi:10.1371/journal.pone.0254920)
Supplement: Attachment — Submitted filename: Response to reviewers Plos one july2ay2.docx [file pone.0254920.s001.docx]

Response to reviewers, PloS One

1. Please ensure that your manuscript meets PLOS ONE's style requirements, including those for file naming. The PLOS ONE style templates can be found at

https://journals.plos.org/plosone/s/file?id=wjVg/PLOSOne_formatting_sample_main_body.pdf and

<https://journals.plos.org/plosone/s/file?id=ba62/PLOSOne_formatting_sample_title_authors_affiliations.pdf>

**Yes, thanks. We have now formatted the titles, etc. to comply with these requirements as well as added acknowledgements and figure titles.**

2. Please provide additional details regarding participant consent. In the ethics statement in the Methods and online submission information, please ensure that you have specified (i) whether consent was informed and (ii) what type you obtained (for instance, written or verbal, and if verbal, how it was documented and witnessed). If your study included minors, state whether you obtained consent from parents or guardians. If the need for consent was waived by the ethics committee, please include this information.

If you are reporting a retrospective study of medical records or archived samples, please ensure that you have discussed whether all data were fully anonymized before you accessed them and/or whether the IRB or ethics committee waived the requirement for informed consent. If patients provided informed written consent to have data from their medical records used in research, please include this information.

**We have added a sentence re: consent.**

Reviewers' comments:

Reviewer's Responses to Questions

Comments to the Author

1. Is the manuscript technically sound, and do the data support the conclusions?

The manuscript must describe a technically sound piece of scientific research with data that supports the conclusions. Experiments must have been conducted rigorously, with appropriate controls, replication, and sample sizes. The conclusions must be drawn appropriately based on the data presented.

Reviewer #1: Yes

**Yes. We agree. However, as there was one question brought up when we published our pre-print that we wanted to address, we added an extra sentence in the methods and results, for completion**.

2. Has the statistical analysis been performed appropriately and rigorously?

Reviewer #1: I Don't Know

3. Have the authors made all data underlying the findings in their manuscript fully available?

The PLOS Data policy requires authors to make all data underlying the findings described in their manuscript fully available without restriction, with rare exception (please refer to the Data Availability Statement in the manuscript PDF file). The data should be provided as part of the manuscript or its supporting information, or deposited to a public repository. For example, in addition to summary statistics, the data points behind means, medians and variance measures should be available. If there are restrictions on publicly sharing data—e.g. participant privacy or use of data from a third party—those must be specified.

Reviewer #1: Yes

4. Is the manuscript presented in an intelligible fashion and written in standard English?

Reviewer #1: Yes

5. Review Comments to the Author

Reviewer #1: The authors Annalee Yassi et al have analysed surveillance data on the infectivity rate among healthcare workers in Vancouver, Canada. Their interpretation was 1) rigorous droplet contact precautions with N95 would prevent occupational infection 2) single dose mRNA vaccination further reduces risk of infection in healthcare workers and that delaying second dose allows widespread vaccination.

The study is quite relevant and interesting. However, I have one querry.

1. The vaccine effectiveness is only 37.2% in HCWs after single dose of mRNA vaccination when compared to unvaccinated HCWs for up to 42 days. They still have risk of contacting the infection. Hence the authors need to mention that second dose is also required for HCWs even if it is delayed.

**Yes, thanks for highlighting this. We have now added a phrase to address the importance of the second dose.**

2. Any idea about the Oxford (AstraZeneca) vaccine effectiveness in the context of HCWs under similar conditions as with mRNA vaccine?

**We had very few HCWs in our cohort who received AstraZeneca (0.1%, 310 individuals). We have added a sentence to the article with this number.**

6. PLOS authors have the option to publish the peer review history of their article (what does this mean?). If published, this will include your full peer review and any attached files.

If you choose “no”, your identity will remain anonymous but your review may still be made public.

Do you want your identity to be public for this peer review? For information about this choice, including consent withdrawal, please see our Privacy Policy.

Reviewer #1: No
